# A Novel Method for Noise Reduction and Jump Correction of Maglev Gyroscope Rotor Signals Under Instantaneous Perturbations

**DOI:** 10.3390/s25072131

**Published:** 2025-03-27

**Authors:** Di Liu, Zhen Shi, Chenxi Zou, Ziyi Yang, Jifan Li

**Affiliations:** 1 School of Geology Engineering and Geomatics, Chang’an University, 126 Yanta Road, Xi’an 710054, China; di.liu@chd.edu.cn (D.L.);; 2 School of Land Engineering, Chang’an University, 126 Yanta Road, Xi’an 710054, China

**Keywords:** maglev gyroscope, external instantaneous perturbations, abnormal jump signal processing, moving average filter (MAF), autoregressive integrated moving average (ARIMA)

## Abstract

The maglev gyroscope torque feedback orientation measurement system, equipped with abundant sampling data and high directional accuracy, plays a crucial role in underground engineering construction. However, when subjected to external instantaneous vibration interference, the gyroscope rotor signal frequently exhibits abnormal jumps, leading to significant errors in azimuth measurement results. To solve this problem, we propose a novel noise reduction algorithm that integrates Moving Average Filtering with Autoregressive Integrated Moving Average (MAF-ARIMA), based on the noise characteristics of the rotor jump signal. This algorithm initially adaptively decomposes the rotor signal, subsequently extracting the effective components of the north-seeking torque with precision and applying MAF processing to effectively filter out noise interference. Furthermore, we utilize the stable sampling trend data of the rotor signal as sample data, employing the ARIMA model to accurately predict the missing abnormal jump trend data, thereby ensuring the completeness and coherence of the rotor signal trend information. Experimental results demonstrate that, compared to the original rotor signal, the reconstructed signal processed by the MAF-ARIMA algorithm exhibits an average reduction of 70.58% in standard deviation and an average decrease of 47.31% in the absolute error of azimuth measurement results. These findings fully underscore the high efficiency and stability of the MAF-ARIMA algorithm in processing gyroscope rotor jump signals.

## 1. Introduction

In the construction of underground projects such as railway tunnels, hydraulic tunnels, and mine tunnels, ensuring the safe and accurate completion of tunneling is a crucial surveying task [1]. Given the complex environment of the tunnels and the numerous limitations imposed by construction conditions, measurement errors in underground direction gradually accumulate as the tunnel extends [2]. This not only increases the risk of tunnel breakthrough but may also result in significant economic losses and serious safety accidents.

The gyroscope total station is a high-precision inertial measurement instrument capable of sensing the directional torque generated by the earth’s rotation and independently determining geographic orientation. It provides a reliable directional reference for tunneling operations [3]. Traditional gyroscope total stations typically employ a suspension band orientation measurement system, which senses directional torque by dynamically tracking the meridian to achieve orientation [4]. However, the pendulum-based system lacks recordable torque information during the orientation process, limiting it to producing only a single orientation measurement result, thereby greatly restricting the reliability and accuracy of the orientation results. To overcome this limitation, the magnetic suspension gyroscope utilizes a torque feedback orientation measurement system for directional measurement [5]. This system applies a counter-torque to the sensitive part of the gyroscope, maintaining it in a torque balance state, and achieves orientation measurement by measuring the magnitude of the counter-torque [6]. Compared to traditional gyroscopes, it provides a more objective and authentic representation of directional torque during the orientation process. By analyzing and refining the sampling signals of the torque sensor, the accuracy and reliability of the orientation results are further optimized, offering engineers a more precise and reliable orientation benchmark for applications such as tunnel penetration measurement.

Additionally, it should be noted that during the orientation measurement process, the sensitive part of the magnetic suspension gyroscope remains in a suspended state, actively isolated from most external vibrations by magnetic suspension. However, due to the gyroscope’s high sensitivity to environmental changes, particularly when there are significant disturbances in the external environment, there may still be residual vibration components that disrupt the torque balance state of the gyroscope, causing abnormal jumps in the gyroscope sampling data. Existing research has demonstrated that rotor signals sampled from the torque sensor rotor system are exceptionally sensitive to environmental changes. The mean of the trend term in these signals reflects the balance position of the gyro’s sensitive part during the north-seeking process [7]. The complexity of the tunnel construction environment, characterized by air flow interference and construction vibrations at the engineering site, often disrupts the gyro torque balance. Instantaneous disturbances in the external environment frequently induce abnormal jumps in the rotor signals, significantly compromising the accuracy of orientation measurement results. Consequently, noise reduction processing of the rotor signals is of paramount importance. Ma Ji conducted an in-depth analysis of multiple sets of rotor signals in complex vibration environments. They introduced an adaptive filtering model based on the optimized wavelet transform (OWT) theory, which effectively enhanced the smoothness of the rotor signals. Furthermore, they utilized high-precision GNSS azimuth angles as constraint conditions to validate the effectiveness of the OWT algorithm [8]. Zhang Xuewei employed the optimized Hilbert–Huang transform (OHHT) method to process rotor signals, reconstructing signal filtering components through Hilbert marginal spectrum analysis technology. This approach successfully reduced high-frequency noise interference [9]. However, both the OWT and OHHT algorithms treat the rotor signal as a whole, thereby struggling to effectively handle jump data within the rotor signals. To mitigate this issue, Wang Yiwen proposed an innovative method that integrates a heuristic segmentation algorithm with the Kolmogorov–Smirnov test (HAS-KS). This method aims to filter out rotor signal sequences affected by instantaneous interference, thereby further enhancing north-seeking accuracy [10]. Although the HAS-KS algorithm has effectively ameliorated abnormal fluctuations in the rotor signals, the impact of noise interference in the retained data and the magnitude changes in the initial data on the orientation measurement results warrant further research and exploration.

To effectively tackle the issue of noise interference in rotor signals stemming from instantaneous environmental vibrations, this study introduces a novel method for reconstructing the rotor jump signals of a magnetic suspension gyroscope. This method integrates the Moving Average Filter (MAF) algorithm with the Autoregressive Integrated Moving Average (ARIMA) model. Initially, the rotor signals are precisely segmented into Stable Sampling Interval (SSI) data and Abnormal Jump Interval (AJI) data, based on their segmented statistical characteristics. Subsequently, the Empirical Mode Decomposition (EMD) algorithm’s adaptive decomposition capability is leveraged to finely decompose the rotor signals, extracting the signal dominant component (SDC) and residual component according to the Hausdorff distance criterion. Here, the SDC primarily captures the subtle changes in north-seeking torque influenced by the earth’s rotation, while the residual component reveals the trend characteristics of the gyroscope’s balance position during the north-seeking process, serving as the trend dominant component (TDC) of the rotor signal. To further mitigate noise interference in the SDC, the study employs the MAF method for in-depth processing, thereby obtaining a denoised signal dominant component. The TDC corresponding to the SSI (TDC_SSI_) is then utilized as sample data to predict the trend information corresponding to the AJI (TDC_AJI_), constructing a complete TDC free from abnormal jump data interference. Finally, by superimposing the denoised SDC with the complete TDC, the denoising and reconstruction of the rotor jump signals are successfully achieved.

To validate the superiority of the MAF-ARIMA algorithm in processing rotor signals with abnormal jumps, the study compares its denoising effects with those of the OWT, OHHT, and HSA-KS algorithms. Experimental results highlight that the MAF-ARIMA algorithm significantly outperforms other algorithms in terms of internal and external consistency accuracy, effectively enhancing the azimuth measurement accuracy of the magnetic suspension gyroscope. This study provides a novel and practical method for the denoising processing of rotor jump signals under the influence of external instantaneous disturbances, offering significant application value in the field of tunnel breakthrough measurement.

## 2. Gyroscope Orientation Measurement Principle and Error Analysis

### 2.1. Torque Balance Orientation Mode of Maglev Gyroscope

**Figure 1:**
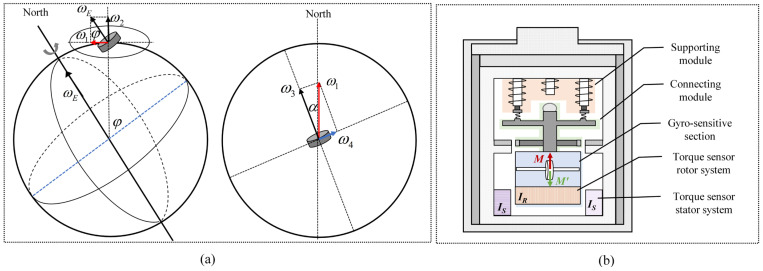
Azimuth measurement principle of the maglev gyroscope and torque feedback measurement system: (**a**) schematic diagram of gyroscope sensing the earth’s rotation components; (**b**) torque feedback measurement system.

Any point on the earth maintains the same rotational angular velocity as the earth, constantly moving from west to east. A high-speed rotating gyroscope is influenced by the equatorial component of the earth’s rotational angular velocity $\omega_{4}$ (Figure 1a), resulting in a directional torque M. The magnitude and direction of this directional torque vary continuously with the earth’s rotation.

(1) $$M=H\times \omega_{E}\cos\varphi \sin\alpha$$

where H is the rotational angular momentum of the gyroscope total station, $\omega_{E}$ represents the rotational angular velocity of the earth, φ represents the local latitude, and α is the gyrocompass north azimuth angle.

To accurately obtain the directional torque, the gyroscopic torque feedback orientation measurement system applies a counter-torque to the gyro sensor section, which is equal in magnitude and opposite in direction to the directional torque. Once the gyro sensor section reaches a state of torque balance, the torque sensor begins to collect rotor signals and stator signals. The magnitude of the gyro counter-torque $M_{i}^{\prime }$ can be calculated based on the sampled data:

(2) $$M_{i}^{\prime }=k\times I_{Ri}\times I_{Si}$$

where k is the torque coefficient, $I_{Ri}$ is the rotor signal, and $I_{Si}$ is the stator signal.

Given the precision and duration required for north azimuth measurements, the instrument automatically collects *N* = 20,000 sets of rotor and stator signals at each equilibrium position. Since each set of rotor and stator signals is obtained through independent observations, we can consider the average torque $M_{mean}$ as the counter-torque at the equilibrium position.

(3) $$M_{mean}=\frac{k\sum_{i=1}^{N}I_{Ri}I_{Si}}{N}$$

Subsequently, based on the torque balance equation $M_{mean}=M$, we can calculate the angle α between the equilibrium position of the gyroscope’s rotation axis and the actual north direction, which is the gyrocompass north azimuth angle [11].

(4) $$\alpha =\arcsin\frac{M_{mean}}{H\omega_{E}\cos\varphi }=\arcsin\frac{k\sum_{i=1}^{N}I_{Ri}I_{Si}}{NH\omega_{E}\cos\varphi }$$

### 2.2. Error Analysis of Rotor Signals Under Instantaneous Vibration Interference

According to the gyroscopic torque balance north-finding principle, the observation data for north-finding primarily consist of rotor signals and stator signals collected by the torque sensor. The operational principle of the torque sensor’s rotor and stator systems primarily relies on electromagnetic induction and the Lorentz force. When the coils in the stator system are energized, they produce a varying magnetic field. This magnetic field interacts with the conductive materials on the rotor, inducing currents within the rotor system. These induced currents then further interact with the magnetic field generated by the stator system, applying a counter-torque to the gyroscope’s sensitive component via the rotor system. By precisely controlling the magnitude and direction of the current in the stator system’s coils, it is possible to accurately regulate the counter-torque, thereby maintaining a torque balance state in the gyroscope’s sensitive part. As illustrated in Figure 1b, the stator system of the torque sensor is securely mounted below the shell, ensuring that it remains in a fixed position throughout the entire north-finding data collection process. This structure guarantees that the stator values remain stable despite changes in the external environment. However, the rotor system of the torque sensor is closely linked to the sensitive part of the gyroscope. It remains in a suspended state during the north-finding data collection period, making it highly sensitive to external vibrations.

During actual observations, due to interference torques caused by complex environments, the counter-torque cannot achieve a perfect balance with the directional torque, resulting in slight oscillations between the sensitive part of the gyroscope and the rotor system. These oscillations introduce noise into the rotor signals collected by the torque sensor, thereby affecting the accuracy of north-seeking.

For magnetic suspension gyroscopes that rely on sensing the earth’s rotation to achieve north-finding, a stable observation environment is fundamental to obtaining high-precision orientation measurement results. Generally, a stable environment does not refer to a specific observation location but rather to an observation environment that simultaneously meets the following two conditions: Firstly, from the perspective of the measurement scenario, it is required that during the orientation measurement process, there are no significant external disturbance sources at the instrument’s setup location, such as air flow disturbances or mechanical vibrations. Secondly, from the perspective of data quality, it is required that the entire north-finding sampling data (typically referring to the rotor signal) does not exhibit significant jumps in its time series.

**Figure 2:**
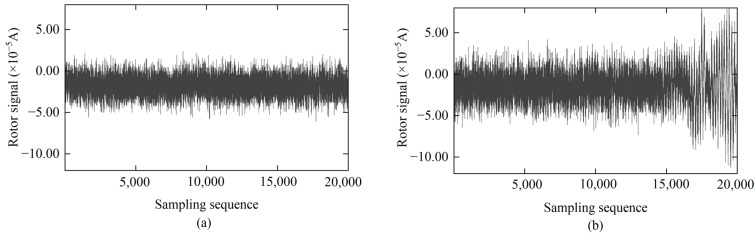
Rotor signal time series under different sampling environments: (**a**) stable environment; (**b**) environment with instantaneous disturbances.

Based on the above description of a stable environment, both enclosed laboratories and open-field environments can be potential stable environments for gyroscopic orientation measurements. For example, the rotor signals corresponding to Figure 2a,b were both collected at the same outdoor observation location. However, the difference is that the rotor signal corresponding to Figure 2a was not affected by external disturbances during the collection process, and its time series demonstrated good mean stability and a low standard deviation. Therefore, the observation environment corresponding to Figure 2a can be considered a stable environment. In a stable environment without significant vibration interference, the collected rotor signals, as shown in Figure 2a, generally exhibit a stable trend but are accompanied by a large amount of white noise and random high-frequency noise. This noise mainly originates from inherent errors in system electronic components, such as analog-to-digital conversion and current–voltage stability, giving the rotor signals a certain bandwidth. The range of the stable signals in Figure 2a is 8.50 × 10^−5^ A, with a standard deviation (SD) of 1.08 × 10^−5^ A, indicating consistency in global and local data characteristics. After averaging a large amount of observation data, there is no shift in the equilibrium position, so the impact on the north-finding results is insignificant.

In contrast, the rotor signals corresponding to Figure 2b, during the collection process, experienced abnormal jumps at the moment that external disturbances occurred due to engineering vehicles passing near the instrument’s setup location. The signals are superimposed with low-frequency amplitude components and high-frequency white noise. This resulted in reduced mean stability and an increased standard deviation. Near 15,000 epochs, the rotor signal experiences significant abnormal fluctuations due to strong instantaneous environmental vibration interference. The range of this set of signals is as high as 2.00 × 10^−4^ A, and the SD increases from 1.45 × 10^−5^ A to 3.03 × 10^−5^ A before and after the vibration, indicating a significant increase in signal dispersion caused by the vibration. More notably, the equilibrium position of the rotor signal also shifts, with the signal mean changing from −1.50 × 10^−5^ A to −1.39 × 10^−5^ A, which directly affects the accuracy of the gyroscope’s north-finding results. Therefore, effective processing and suppression of noise in the rotor signal under vibration environments must be carried out.

## 3. Methodology

### 3.1. Segmentation of Rotor Signal Intervals

To accurately capture the rotor of occurrence and termination of data jumps, we process the rotor signals via segmentation. Previous studies have shown that after being segmented approximately 20 times [10], the rotor signal can effectively reveal the statistical characteristics of abnormal jump data. Based on this finding, we chose 1000 epochs as the window length to segment the rotor signal. Equation (4) indicates that the mean value of the rotor signal significantly affects the measurement result of the north azimuth angle. Therefore, we regard the change in mean stability as one of the critical indicators for identifying jump phenomena. Moreover, the SD, as an essential index for evaluating the dispersion of data, effectively complements the deficiency of the mean value in measuring signal fluctuations. Therefore, in this study, we use the mean value and the SD of segmented data as core indicators to precisely identify the occurrence and termination of abnormal jumps in a rotor signal.

For a rotor signal that exhibits a jump, we employ a sequential method to analyze the statistical characteristics of the window data during the process of determining the moment when the abrupt change occurs. During this process, we identify the maximum window index at which both the mean and SD exhibit an abrupt change, designating this as the initial window of the jump (non-steady-state window). However, there is an obvious theoretical flaw in directly identifying the moment of abrupt statistical feature change in the window data as the starting moment of the abrupt change. This is because the resolution of the rotor signal window is set to 1000 sampling points, and when the disturbed signal within the window is below the resolution threshold, its statistical features may still appear stable. This pseudo-steady-state phenomenon can lead to misjudgment of the actual moment of abrupt change. To ensure that the sampling interval completely excludes transient interference, we extend the non-steady-state window forward by one unit length as a metastable window based on the determined window sequence number corresponding to the obvious abrupt change. Equation (5) illustrates the final determined moment of abrupt change. The mechanism of moving the window forward from the starting moment of the abrupt change effectively avoids the metastable region at the edge of the window by establishing a safety boundary.

(5) $$T_{\mathrm{start}}=\max(W_{m1},W_{s1})+1$$

where $T_{\mathrm{start}}$ is the window index corresponding to the rotor signal when the jump occurs. $W_{m1}$ and $W_{s1}$ are the window indices of the rotor signal when the mean and SD of the sequentially segmented rotor signal data undergo a sudden change.

In the process of ascertaining the termination position of a jump in the rotor signal, we adopt a reverse-sequential approach to analyze the statistical characteristics of the window data. Consistent with the methodology for determining the starting moment, we extend the non-steady-state termination window by one unit length to effectively avoid the metastable window. Furthermore, considering that the rotor system only undergoes data sampling under strictly stable conditions, the reconstruction process of the instrument’s torque balance state exhibits a time-lag characteristic after the elimination of external disturbances. Based on the statistical analysis of a substantial amount of experimental data related to the establishment of stable states in instruments, the results indicate that the system can complete dynamic balance recovery within two window periods (the time-lag window). Therefore, as shown in Equation (6), the final algorithm comprehensively extends the non-steady-state window at the end of the abrupt change backward by two units, taking the larger value between the metastable window and the time-lag window. This window expansion mechanism for the termination moment of the abrupt change not only mitigates the risk of misjudgment due to resolution limitations but also reserves sufficient time-domain for the system’s dynamic recovery.

(6) $$T_{\mathrm{end}}=\max(W_{m2},W_{s2})+2$$

where $T_{\mathrm{end}}$ is the window index corresponding to the rotor signal when the jump ends. $W_{m2}$ and $W_{s2}$ are the window indices of the rotor signal when the mean and SD of the rotor signal, segmented in reverse order, undergo a sudden change.

The time-window correction mechanism proposed in this study is fundamentally grounded in a coupled analysis of the physical constraints inherent in the signal acquisition system and the system’s dynamic characteristics. During the initial stage of an abrupt change, window resolution serves as the primary constraint. Conversely, in the termination stage of the abrupt change, both the resolution limitation and the system’s dynamic response must be considered concurrently. This differentiated treatment strategy not only ensures the accurate capture of transient processes but also accommodates the system’s inherent dynamic characteristics, ultimately leading to the differentiated offset design evident in Equations (5) and (6). Based on Equations (5) and (6), we achieved precise localization of the jump data in the rotor signal. We further categorized the rotor signal into SSI data and AJI data for subsequent targeted processing.

### 3.2. Noise Processing Methods for Decomposed Components of Rotor Signals

#### 3.2.1. Adaptive Decomposition of Rotor Signals and Extraction of Effective Components

EMD is a signal decomposition method based on the local characteristics of the signal [12]. This method can gradually decompose the different scales or trend components that genuinely exist in the rotor signal into a series of independent intrinsic mode functions (IMFs) and a TDC [13]. After the EMD processing of any target signal x(t), the following results are obtained:

(7) $$x(t)=\sum_{i=1}^{n}c_{i}(t)+r_{n}(t)$$

where $c_{i}(t)$ is the intrinsic mode function and $r_{n}(t)$ is the residual component, which represents the TDC of x(t).

After applying EMD processing to the rotor signal, we obtain a series of IMFs and a TDC, as shown in Equation (7). To accurately measure the similarity between the IMFs and x(t), we employ the Hausdorff distance determination method [14], which has been widely used in fields such as signal denoising and fault diagnosis [15,16,17]. The Hausdorff distance, by quantifying the maximum geometric deviation between two points sets, offers a unique mechanism for measuring non-corresponding differences. In traditional signal component analysis methods, commonly used similarity measures include the Euclidean distance, mean squared error, and cross-correlation coefficient. However, these methods all exhibit significant limitations in noisy scenarios: the Euclidean distance requires a strict point-by-point correspondence, and its results are easily disturbed by noise points; the mean squared error, by averaging squared differences, may weaken extreme discrepancies; the cross-correlation coefficient is highly sensitive to signal phase and requires strict alignment of signal lengths. In contrast, the Hausdorff distance, by calculating the bidirectional maximum minimum distance, focuses on capturing the worst-case matching deviation between two points sets, demonstrating robustness to local noise, non-corresponding measurement, and geometric sensitivity, thereby effectively capturing the structural differences in signal components. Considering the unique noise characteristics of rotor signal components, which manifest as the superposition of global white noise and local high-frequency noise, we have selected the Hausdorff distance as the discriminant criterion. This measure, compared to traditional methods, more robustly reflects the intrinsic differences among signal components, thereby effectively avoiding misjudgments due to noise interference. In this study, we use the index *h* of the first maximum point in the Hausdorff distance sequence as the boundary, based on which the IMFs are divided into noise-dominated components (NDCs) and SDCs [18], thus determining the properties of the signal decomposition results.

(8) $$c(t)=\sum_{i=1}^{h}c_{i}(t)+\sum_{i=h+1}^{n}c_{i}(t)=\mathrm{NDC}+\mathrm{SDC}$$

The NDC mainly contains high-frequency noise interference introduced by instantaneous vibrations from the external environment, while the SDC primarily comprises the north-finding information collected by the torque sensor rotor system. To improve the signal quality, as shown in Equation (9), we adopt a strategy of eliminating the NDC and retaining the SDC, thus achieving preliminary noise reduction processing for x(t).

(9) $$\hat{x}(t)=\sum_{i=h+1}^{n}c_{i}(t)+r_{n}(t)=\mathrm{SDC}+\mathrm{TDC}$$

#### 3.2.2. Noise Reduction Method for Rotor SDCs Based on MAF

During the process of decomposition of the original rotor signal, we eliminated the noise-dominated components, which were primarily high-frequency, based on the Hausdorff distance criterion. However, the obtained SDC still suffered from interference caused by low-frequency noise components, resulting in significant fluctuations. To solve this issue, this study further employs the MAF algorithm to denoise the SDC, aiming to effectively smooth the data sequence, substantially reduce random fluctuations, and enhance the quality of the signal.

The MAF algorithm is a classic technique in the field of digital signal analysis, attracting attention for its excellent smoothing capability, efficient random fluctuation suppression performance, and trend highlighting ability [19,20]. This algorithm achieves robust filtering effects by calculating the average of data points and their neighboring points, while also possessing significant advantages such as simplicity of the algorithm, rapid real-time response, and high smoothness [21]. The core concept of the MAF algorithm lies in performing local averaging of data through a sliding window. In practice, for a given time series data, a fixed window size is first determined. The algorithm then iterates through the data point by point, calculating the average of the data within each window, and assigns this average as the filtered output for the current data point. As the window slides incrementally, the filtered result for the entire data sequence is ultimately generated. The detailed computational process of the moving average filtering algorithm can be described by Equation (10).

(10) $$y(t_{n})=\frac{1}{s}\sum_{i=n-(s-1)}^{n}x(t_{i})$$

where s is the length of the sliding filter window, $x(t_{i})$ is the i-th sample value, and $y(t_{n})$ is the n-th measured value obtained from s sample values.

In the MAF algorithm, the selection of window size is particularly crucial. If the window is too small, the filtering effect will be difficult to manifest; conversely, if the window is too large, it may lead to excessive smoothing of the signal, even resulting in the loss of key signal characteristics. To ensure accurate identification of the jump position, we set the window size of the MAF algorithm based on the sampling frequency of the rotor signal, which is 200. Furthermore, although the MAF algorithm has evolved to include weighted optimizations such as the weighted moving average [22] and exponential moving average [23] with the expansion of application fields, considering that the rotor signal processed in this paper maintains independence at each data point within the window during the acquisition process, the default equal-weight moving average algorithm is ultimately chosen for denoising the rotor signal within the window.

#### 3.2.3. Correction Processing of SDC Noise Reduction Results

To ensure the consistency of data continuity and statistical characteristics across different sampling intervals, it is necessary to perform correction processing on the SDC-denoised results corresponding to the AJI (SDC_AJI_). This includes extreme value normalization and mean compensation. If only one SSI is adjacent to the AJI data in the original rotor signal, the denoised results of the SDC_AJI_ will be corrected based on the SDC-denoised results corresponding to that SSI (SDC_SSI_). However, if two SSIs are adjacent to the AJI data, we establish the principles of extreme value normalization and mean compensation based on the SD, as shown in Equations (11) and (12), respectively.

(11) $$\left\{\begin{array}{l} u=\frac{\max({\mathrm{SDC}}_{\mathrm{SSI}\text{-}1,2}^{\prime })-\min({\mathrm{SDC}}_{\mathrm{SSI}\text{-}1,2}^{\prime })}{\max({\mathrm{SDC}}_{\mathrm{AJI}}^{\prime })-\min({\mathrm{SDC}}_{\mathrm{AJI}}^{\prime })}\\ {\mathrm{SDC}}_{\mathrm{AJI}\text{-}n}^{\prime }=\min({\mathrm{SDC}}_{\mathrm{SSI}\text{-}1,2}^{\prime })+u\times ({\mathrm{SDC}}_{\mathrm{AJI}}^{\prime }-\min({\mathrm{SDC}}_{\mathrm{AJI}}^{\prime })) \end{array}\right.$$

where ${\mathrm{SDC}}_{\mathrm{SSI}\text{-}1,2}^{\prime }$ is the noise reduction result of the two SDC_SSI_, ${\mathrm{SDC}}_{\mathrm{AJI}}^{\prime }$ is the noise reduction result of SDC_AJI_, and ${\mathrm{SDC}}_{\mathrm{AJI}\text{-}n}^{\prime }$ is the normalized extreme value result of ${\mathrm{SDC}}_{\mathrm{AJI}}^{\prime }$.

(12) $$\left\{\begin{array}{l} mc=\frac{{\mathrm{SDC}}_{\mathrm{SSI}\text{-}1}^{\prime }\times std_{2}+{\mathrm{SDC}}_{\mathrm{SSI}\text{-}2}^{\prime }\times std_{1}}{std_{1}+std_{2}}\\ {\mathrm{SDC}}_{\mathrm{AJI}\text{-}m}^{\prime }={\mathrm{SDC}}_{\mathrm{AJI}\text{-}n}^{\prime }+(mc-{\mathrm{SDC}}_{\mathrm{AJI}\text{-}n}^{\prime }) \end{array}\right.$$

where $std_{1}$ is the SD of ${\mathrm{SDC}}_{\mathrm{SSI}\text{-}1}^{\prime }$, $std_{2}$ is the SD of ${\mathrm{SDC}}_{\mathrm{SSI}\text{-}2}^{\prime }$, and ${\mathrm{SDC}}_{\mathrm{AJI}\text{-}m}^{\prime }$ is the mean compensation result of ${\mathrm{SDC}}_{\mathrm{AJI}\text{-}n}^{\prime }$.

### 3.3. Prediction and Fusion of Rotor Signal Trend Data

The impact of instantaneous vibrations can lead to significant errors in the TDC_AJI_, resulting in a deviation in the mean value of the residual component. Considering that the mean value of the rotor signal is mainly determined by the TDC obtained through EMD, we effectively avoid the noise caused by instantaneous vibrations on the mean value of the TDC by eliminating the TDC_AJI_. However, this operation inevitably leads to missing data in the TDC. To ensure the integrity of the TDC, we also need to predict the missing data accurately.

#### 3.3.1. ARIMA Prediction Model

The ARIMA model is one of the most important and widely used time series models [24]. The basic model of the autoregressive moving average model is ARMA (*p*, *q*), and its expression is

(13) $$Y_{t}=\varphi_{1}Y_{t-1}+\varphi_{2}Y_{t-2}+\cdots +\varphi_{p}Y_{t-p}+\varepsilon_{t}-\theta_{1}\varepsilon_{t-1}-\theta_{2}\varepsilon_{t-2}-\cdots -\theta_{q}\varepsilon_{t-q}$$

where $Y_{t}$ is the predicted time series, φ is the AR model parameter, θ is the MA model parameter, ε is zero-mean white noise, p is the number of autoregressive terms, and q is the time index.

Notably, the ARMA model is suitable for stationary time series. In contrast, the ARIMA model adds differencing transformations to the ARMA model, which transforms nonstationary time series, making them more suitable for analysis using the ARIMA model. Generally, the ARIMA prediction model can be simplified as ARIMA(*p*, *d*, *q*) [25], and its specific expression is

(14) $$\varphi_{p}(B){\left(1-B\right)}^{d}Y_{t}=\theta_{q}(B)\varepsilon_{t}$$

where B is the backward shift operator satisfying the relation $BY_{t}=Y_{t-1}$, and d is the number of differencing terms.

In time series prediction, the ARIMA model is applied based on the assumption that the sample data are stationary. The Augmented Dickey–Fuller (ADF) test and the Kwiatkowski–Phillips–Schmidt–Shin (KPSS) test are two pivotal methods in time series analysis for determining signal stationarity. These tests assess the stationarity of rotor signals by examining the presence of a unit root and testing for stationarity or trend stationarity, respectively, which aids in determining appropriate parameters for the ARIMA model. However, while the ADF test is proficient at detecting unit root non-stationarity (such as random walks), it may be ineffective for trend-stationary sequences that require detrending. Conversely, the KPSS test is adept at identifying trend non-stationarity (such as deterministic trends) but may lack sensitivity to unit root non-stationarity. Therefore, in this study, we introduce a composite indicator known as the C_AK_ test method for rotor signal stationarity assessment, which enhances the robustness of the judgment through a dual-verification mechanism. By integrating the ADF and KPSS tests and employing complementary hypothesis logic, we improve the reliability of signal stationarity assessment, effectively distinguishing between unit root non-stationarity, trend stationarity, and deterministic trends, thereby mitigating the limitations of relying on a single test [26,27,28]. In this study, we set clear judgment criteria: if the C_AK_ test result is 1, then the sample data are stationary and satisfy the application conditions of the ARIMA model; if the C_AK_ test result is not 1, then the sample data are nonstationary, and the model parameter d needs to be adjusted.

In addition, the AIC and BIC are vital indicators for determining the *p* and *q* parameters in the ARIMA model [29,30]. In this study, we fully consider the advantages of the AIC in preventing underfitting and the role of the BIC in preventing overfitting. Therefore, we propose using the sum of the AIC and BIC as a new information criterion called the $C_{\mathrm{IC}}$. This ensures that the selected model has a good fit, further enhancing the accuracy and reliability of the time series predictions.

(15) $$C_{\mathrm{IC}}=2k+k\log(n)-4(\log L)$$

where logL is the likelihood of the model, k is the number of model parameters, and n is the number of observations in the time series data.

#### 3.3.2. Bilateral Linear Weighted Fusion of Prediction Results

Similarly to the scenario described in Section 3.2.3, when the AJI data is adjacent to only one SSI, we directly use the prediction result of that SSI data as the final prediction result. However, when the AJI is adjacent to two SSIs, we propose a linear weighting approach, as the prediction accuracy tends to decrease with increasing prediction length, as shown in Equation (16). This method can effectively fuse the prediction results of two adjacent SSIs, thereby improving the overall prediction accuracy.

(16) $$P_{j}=P_{1,j}\times (1-\frac{j-1}{m-1})+P_{2,j}\times \frac{j-1}{m-1},j\in [1,m]$$

where $P_{1,j}$ is the prediction result obtained using TDC_SSI-1_ as the sample data and $P_{2,j}$ is the prediction result obtained using TDC_SSI-2_ as the sample data, where m is the length of the missing TDC.

**Figure 3:**
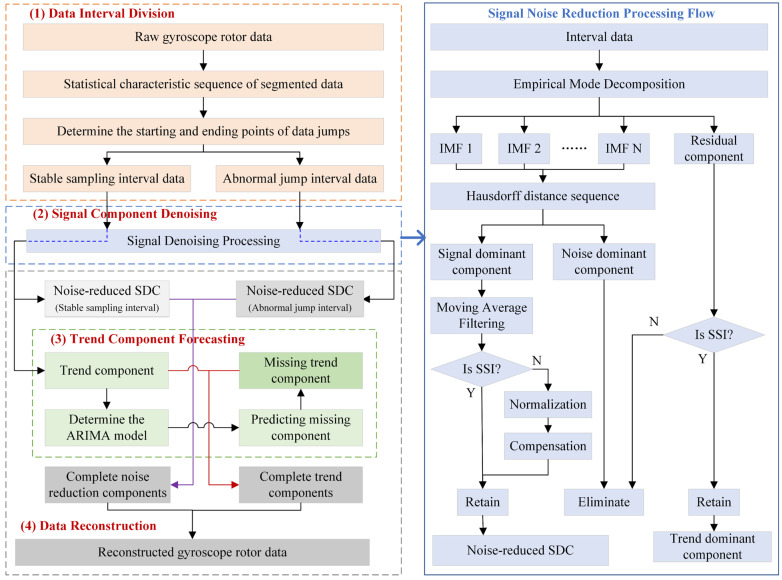
Processing flow of jumping rotor signals based on the MAF-ARIMA algorithm.

To address the issue of sampling data jumps in the maglev gyroscope caused by instantaneous vibration interference, thereby compromising the instrument’s north-finding accuracy, we have developed a reconstruction algorithm for handling rotor jump signals. The overall flowchart of the proposed MAF-ARIMA algorithm is presented in Figure 3.

## 4. Results Analysis and Discussion

### 4.1. Experimental Data Source and Evaluation Indicators

**Figure 4:**
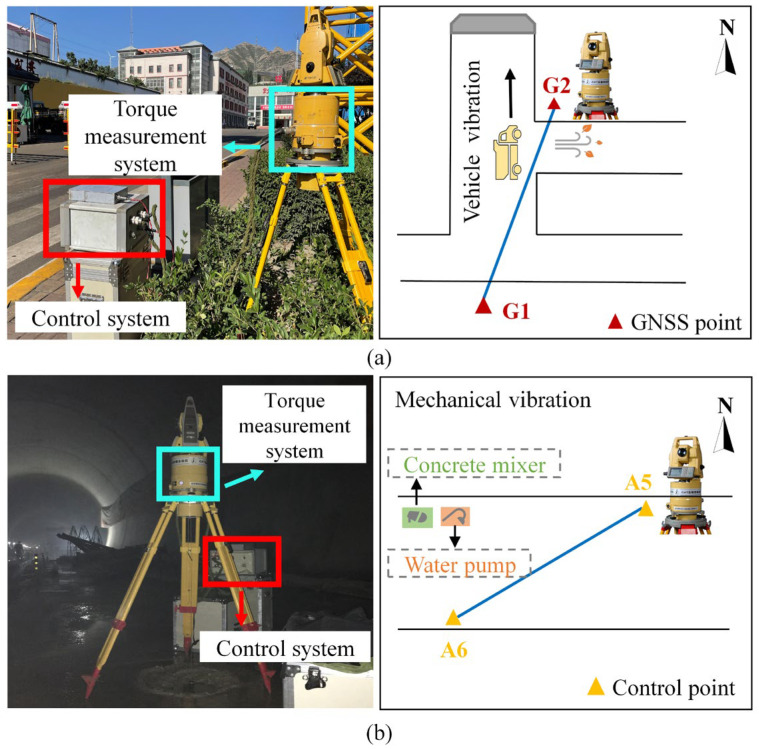
Schematic diagram of on-site experimental scenes and transient interference sources: (**a**) Tashan Coal Mine; (**b**) Longnan Tunnel.

To evaluate the performance of the algorithm, we conducted directional measurement experiments at two locations: the Tashan Coal Mine, located in Datong city, Shanxi Province, China, as shown in Figure 4a, and the Longnan Tunnel, a section of the Beijing–Hong Kong High-Speed Railway in Jiangxi-Shenzhen, as depicted in Figure 4b. The coal mining tunnel of Tashan Coal Mine has a total length of 16 km. The thickness and development of coal seams and rock strata vary significantly, and numerous uncertain factors, such as faults and collapse columns, significantly increase the difficulty of tunnel connection [31]. The Longnan Tunnel, with a total length of 10.24 km, traverses 12 fault and fracture zones, representing a challenging and high-risk control project for the entire Jiangxi–Shenzhen High-Speed Railway [32].

As shown in Figure 4, during the field experiments, the torque sensor rotor system was subjected to instantaneous vibration interference generated by vehicles, wind, and construction machinery, resulting in abnormal jumps in the rotor signals. We collected five sets of rotor signals containing abnormal jumps at each experimental site by applying four noise reduction algorithms, OWT, OHHT, HSA-KS, and MAF-ARIMA, to process the ten sets of rotor signals. We also verified the internal and external accuracy of the denoising results, thoroughly demonstrating the effectiveness and stability of the proposed algorithm in this study.

The SD is a crucial indicator commonly used in statistics to assess the dispersion of a dataset. It effectively quantifies the degree of variation of each value $s_{i}$ in the dataset from the mean value $s_{mean}$. A slight SD indicates a relatively low dispersion of the dataset, meaning that the sampling data are more concentrated. In this experiment, we adopt the SD as the evaluation criterion for internal accord accuracy, according to Equation (17), to compare the effects of different noise reduction methods.

(17) $$\mathrm{SD}=\sqrt{\sum_{i=1}^{n}(s_{i}-s_{mean}{)}^{2}/n-1}$$

The ultimate goal of denoising the rotor signal is to improve the azimuth measurement accuracy. Therefore, we use the absolute error (D) as an evaluation metric for the external accord accuracy. In this experiment, the instrument setup points were located within a high-precision GNSS control network or a completed tunnel control network. The directional accuracy of the control network is better than 0.5″, which significantly exceeds the nominal accuracy of the instrument, which is 3.5″. Therefore, in this experiment, we consider the north azimuth $\alpha_{0}$ of the control network as the true value. After denoising the rotor signal, we substitute the denoised data into Equation (4) to obtain the corresponding azimuth measurement result α. Then, we can calculate the D value between α and the true north azimuth using Equation (18). The smaller the D value is, the more significant the denoising effect.

(18) $$D=\left|\alpha^{*}-\alpha_{0}\right|$$

### 4.2. Processing Results of the Jumping Rotor Signal

After analyzing the ten sets of experimental data, we found that abnormal jumps commonly occur in the middle or at the endpoints of the rotor signals. Considering that the rotor signal with midrange jumps can be regarded as the superimposed effect of two endpoint jump signals, in Section 4.2.1, we use a set of rotor signals with midrange jumps as an example to discuss the signal denoising process in detail. Additionally, in Section 4.2.2, we explain the denoising results for the ten sets of rotor signals.

#### 4.2.1. Processing Results of the Rotor Signals with Midrange Jumps

**Figure 5:**
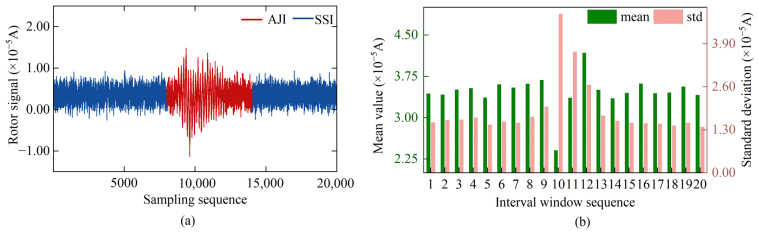
Jumping rotor signal under the influence of instantaneous disturbance: (**a**) time series of the rotor signal; (**b**) statistical characteristics of the segmented data of the rotor signal.

During the data sampling process, rotor signals exhibit significant oscillation phenomena due to external instantaneous vibration interference, as shown in Figure 5a. This oscillation phenomenon leads to sudden changes in the SD and mean value of the segmented data corresponding to the jumps, as depicted in Figure 5b. However, once these external interferences disappear, the instrument re-establishes a stable state, and the abnormal fluctuations in the rotor signal also subside. Based on the calculation results of Equations (5) and (6), we determined that the abnormal jump interval in the rotor signal is between epochs 8001–14,000, while epochs 1–8000 represent the first SSI (SSI-1), and epochs 14,001–20,000 represent the second SSI (SSI-2).

**Figure 6:**
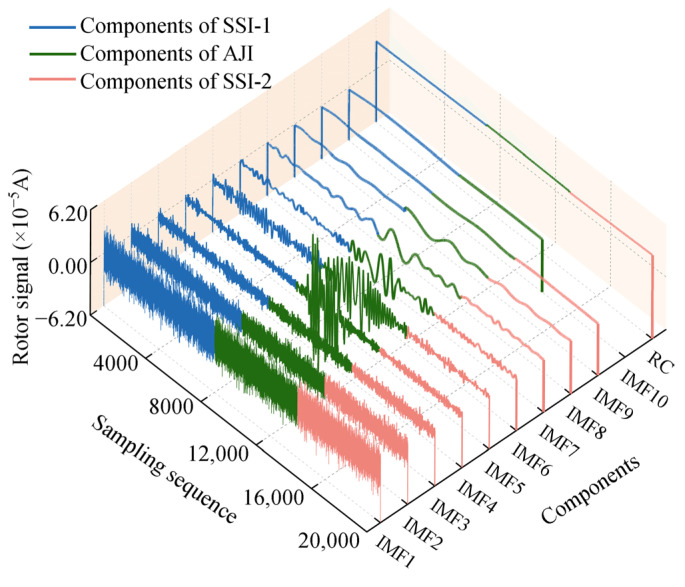
Adaptive decomposition results of rotor signals processed by EMD method.

**Figure 7:**
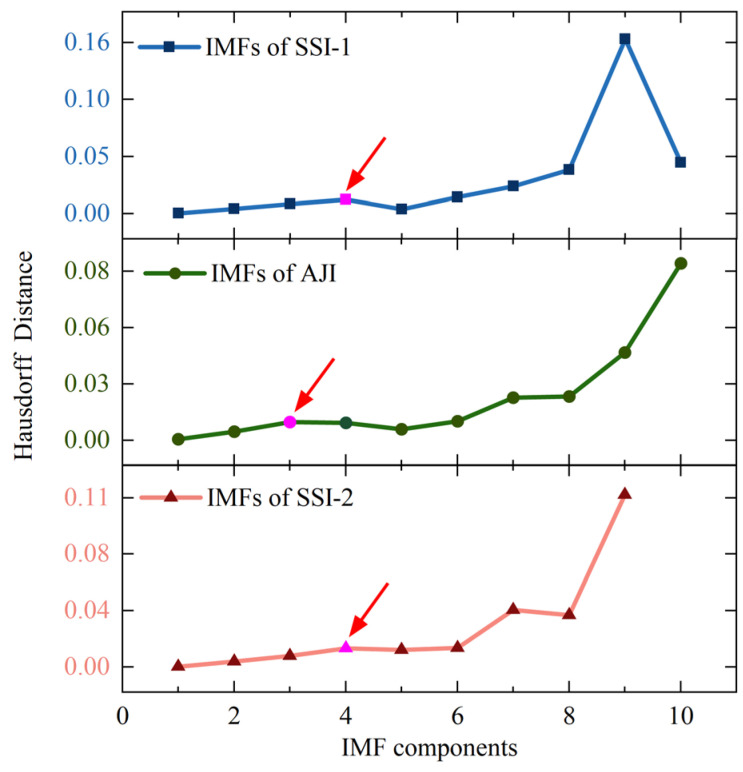
The sequence of Hausdorff distances between IMFs and the original rotor signal within different sampling intervals.

**Figure 8:**
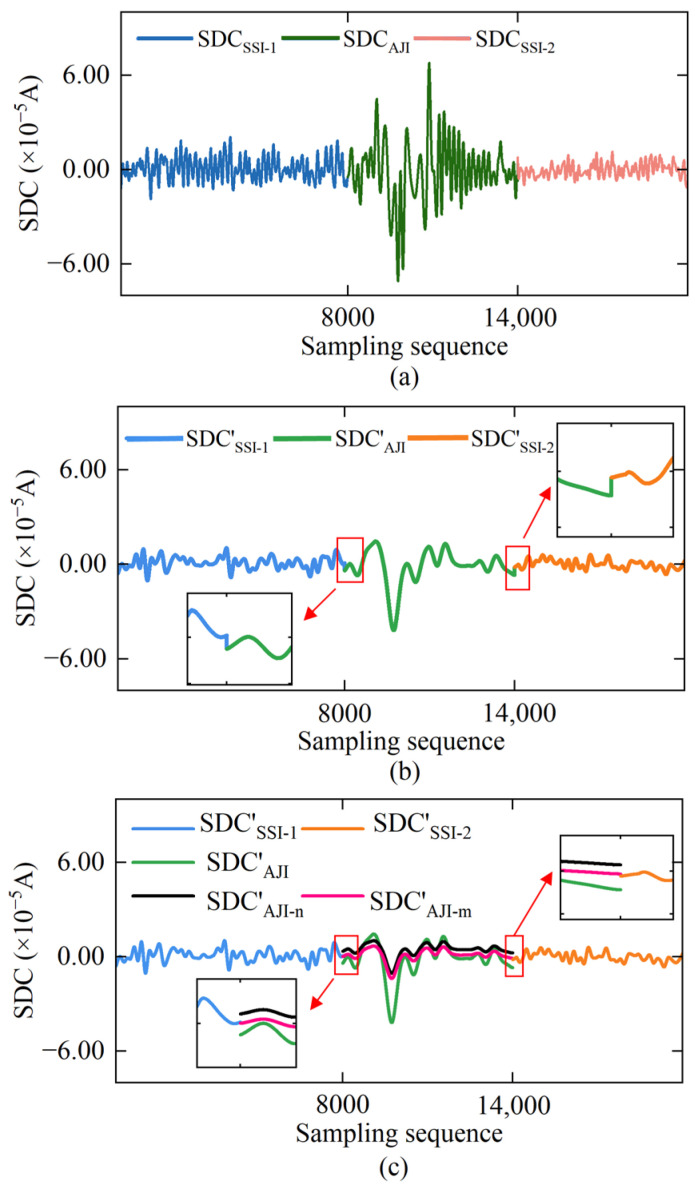
Extraction results of SDC and noise reduction correction in rotor signal: (**a**) SDC extraction result; (**b**) noise reduction result of SDC processed by MAF method; (**c**) extreme value normalization and mean compensation for SDC_AJI_ noise reduction result.

The results of processing rotor signal data in each sampling interval using the EMD method are shown in Figure 6. After decomposition, each sampling interval of the data corresponds to a series of IMFs with gradually decreasing frequencies and a TDC. By referring to the Hausdorff distance sequence presented in Figure 7, we can identify IMF1-IMF4, IMF1-IMF3, and IMF1-IMF4 as the high-frequency noise mode components corresponding to SSI-1, AJI, and SSI-2, respectively. Subsequently, based on Equation (8), we eliminated these high-frequency mode components and reconstructed the remaining IMFs by superposition, obtaining the SDC, as depicted in Figure 8a, thus achieving initial noise reduction of the rotor signal. Comparing Figure 5a with Figure 8a, it can be observed that the initial noise reduction effectively eliminates external high-frequency noise interference while preserving the detailed characteristics of the original signal well.

The result of the MAF applied to the SDC is shown in Figure 8b. Compared to Figure 8a, the noise interference in the SDC is further eliminated, and the spike phenomenon in the denoised signal is significantly reduced. In addition, the discrete fluctuations within the AJI data have also been notably improved. However, due to the difference in the data fluctuation amplitude between the AJI and SSI, as well as the different processing parameter settings for each sampling interval, signal discontinuity occurs at the data connection points (8000 epochs, 14,000 epochs), as shown in Figure 8b. To address this issue, we applied extreme value normalization and mean compensation to the SDC_AJI_ denoising results based on Equations (11) and (12). The corrected results are presented in Figure 8c, where the discrete fluctuation amplitude within the AJI data is significantly reduced, and the continuity and mean stability of the rotor signal across the entire sampling interval are also ensured.

**Figure 9:**
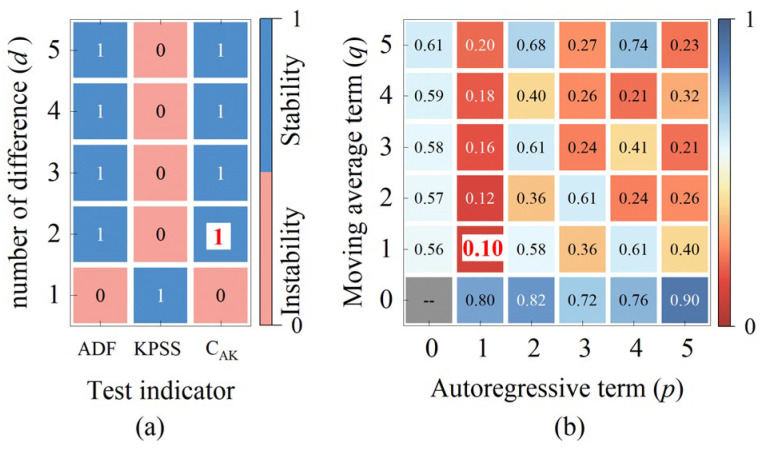
The results of ARIMA prediction model parameters based on TDC_SSI-1_ sample data: (**a**) the process of determining the number of differencing term *d*; (**b**) the process of determining the number of autoregressive term *p* and the time index *q*.

**Figure 10:**
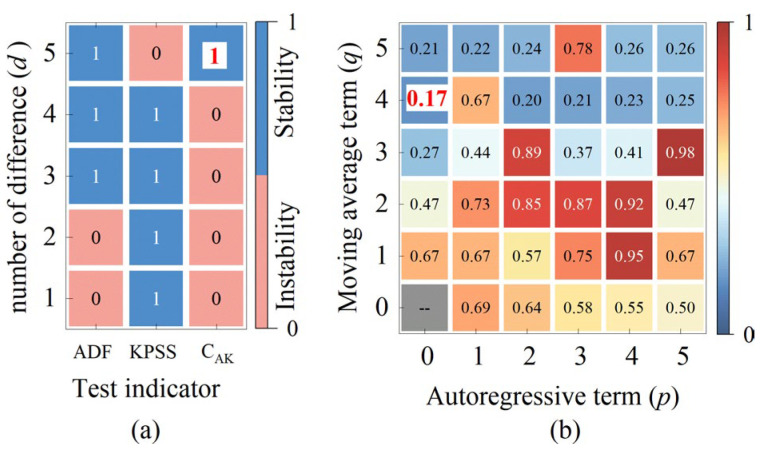
The results of ARIMA prediction model parameters based on TDC_SSI-2_ sample data: (**a**) the process of determining the number of differencing term *d*; (**b**) the process of determining the number of autoregressive term *p* and the time index *q*.

**Figure 11:**
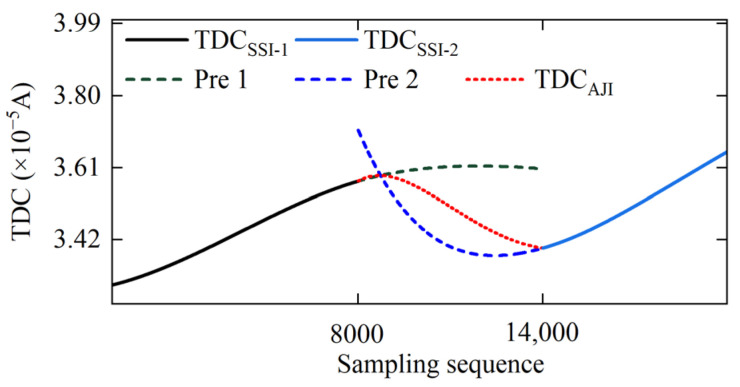
The TDC_AJI_ prediction results corresponding to different sample data and the fusion processing of these prediction results.

The process of determining the parameters for ARIMA prediction model of the TDC_AJI_ is illustrated in Figure 9 and Figure 10. Figure 9a demonstrates that after applying a second-order difference to TDC_SSI-1_, the C_AK_ hypothesis is 1, indicating that the current differenced data already satisfy the requirement of signal stationarity. Furthermore, Figure 9b reveals that when *p* = 1 and *q* = 1, the information criterion C_IC_ reaches its minimum value. Based on these parameter settings, we constructed an ARIMA(1, 2, 1) prediction model and used TDC_SSI-1_ as the sample data to predict the TDC_AJI_. The prediction results are presented in Figure 11 (Pre 1). Similarly, for TDC_SSI-2_, when the order of differencing *d* = 5, the data meet the stationarity requirement. Furthermore, when *p* = 0 and *q* = 4, the information criterion again attains its minimum value. Accordingly, we constructed an ARIMA(0, 5, 4) prediction model and used the TDC_SSI-2_ as the sample data to predict the TDC_AJI_. The prediction results are shown in Figure 11 (Pre 2). Since the prediction accuracy typically decreases gradually with increasing prediction length, we weighted and fused the forward prediction result Pre 1 and the backward prediction result Pre 2 according to Equation (16). The final fused prediction result of the TDC_AJI_ is shown in Figure 11 (TDC_AJI_).

**Figure 12:**
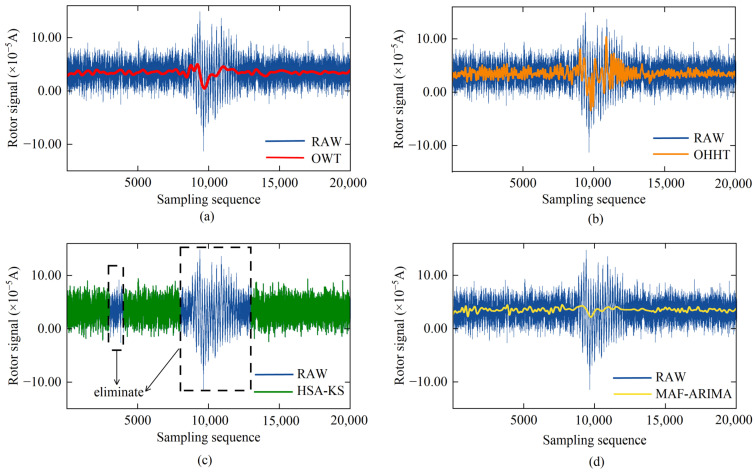
Noise reduction results of rotor signals using different algorithms: (**a**) OWT; (**b**) OHHT; (**c**) HSA-KS; (**d**) MAF-ARIMA.

**Figure 13:**
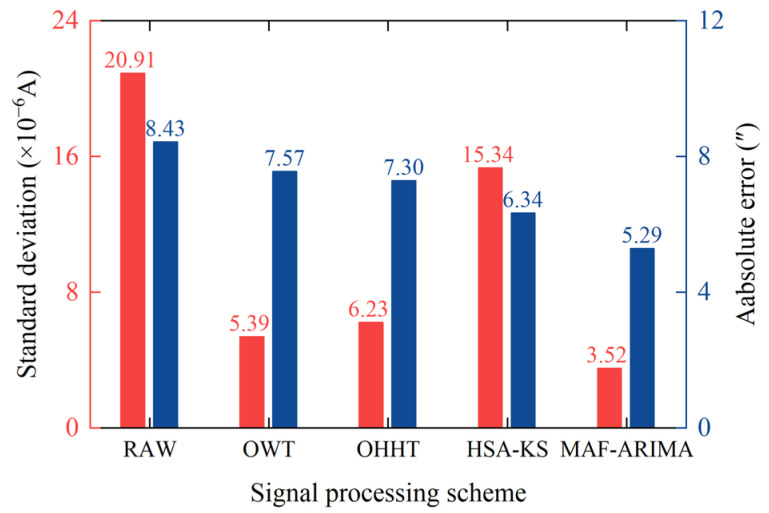
Comparison of noise reduction effects.

The SDC denoising result shown in Figure 8c is overlaid with the complete TDC shown in Figure 11 to obtain the reconstructed rotor signal processed by the MAF-ARIMA method, as illustrated in Figure 12d. To comprehensively evaluate the accuracy of the reconstructed signal, we further analyzed its internal and external consistency accuracy, and the results are presented in Figure 13. It is evident from Figure 13 that the reconstructed signal processed by the MAF-ARIMA method exhibits significant advantages compared to those processed by the other three methods. Its SD is only 3.52 × 10^−6^ A, indicating that the data fluctuation of this reconstructed signal is minimal throughout the entire signal sampling interval. Moreover, the D value of the reconstructed signal processed by MAF-ARIMA significantly decreases from the original 8.43″ to 5.29″. This suggests that this method also effectively suppresses abnormal data jumps caused by transient vibrations.

After processing with the OWT method, the reconstructed rotor signal is shown in Figure 12a. We can see a significant reduction in the SD of the reconstructed signal, indicating that setting a higher wavelet decomposition level can significantly improve the overall smoothness of the reconstructed signal, reducing its SD to 5.39 × 10^−6^ A. However, since the OWT method targets the entire rotor signal for noise reduction, it is ineffective in handling local abnormal jumps in the sampled signal. Therefore, there are still relatively obvious abnormal fluctuations in the reconstructed signal. Specifically, the D value of the reconstructed signal is only reduced by 0.86″, reflecting the significant limitations of this method in reducing the interference caused by instantaneous vibrations on the torque sensor.

The reconstructed rotor signal after processing with the OHHT method is presented in Figure 12b. The removal of high-frequency noise modal components from the IMFs has significantly enhanced the smoothness of the reconstructed signal, reducing its SD to 6.23 × 10^−6^ A. However, the inherent limitations of the EMD method, such as mode mixing, pose challenges in completely separating high-frequency noise from low-frequency signals. Consequently, despite the decrease in SD, the reconstructed signal still contains numerous spikes, which compromise its purity. In terms of noise reduction effectiveness, the D value associated with the EMD method has been reduced from 8.43″ to 7.30″, representing a tangible improvement over the OWT method. Nevertheless, the persistence of data fluctuations within the AJI in both the SDC and TDC means that the enhancement in the accuracy of the reconstructed signal is relatively modest.

The reconstructed rotor signal processed by the HSA-KS method is shown in Figure 12c. This method identifies and directly removes 6000 jump data points from the raw rotor signal, effectively minimizing noise interference. As the removed data mainly consist of external noise components, the reconstructed signal’s noise reduction performance is significantly enhanced compared to the OWT and OHHT methods, with the D value decreasing to 6.34″. Nevertheless, the HSA-KS method does not address the data within the SSI, leading to no significant improvement in the internal conformance accuracy of the reconstructed signal. The signal’s dispersion remains substantial, with an SD of 15.34 × 10^−6^ A. Additionally, the decreased volume of initial data required for computing the gyroscope reverse torque might explain why this method’s noise reduction effectiveness is not on par with that of the MAF-ARIMA method.

#### 4.2.2. Statistical Results of Noise Reduction for Sampling Data in the Experimental Area

After conducting noise reduction processing on ten sets of rotor signals containing abnormal jumps, we obtained detailed results, as shown in Table 1 and Table 2. Through comparative analysis, it can be found that all four methods can improve the abnormal jumps in the rotor signals and reduce the interference of instantaneous vibrations on the torque sensor sampling signals.

The experimental results show that the noise reduction effects of the OWT and OHHT methods are comparable. After processing with the OWT and OHHT methods, the average SD of the reconstructed signals decreased from 18.49 × 10^−6^ A to 7.36 × 10^−6^ A and 10.44 × 10^−6^ A, respectively, indicating a significant improvement in the dispersion of the rotor signals. However, the D value gain rates of the OWT and OHHT methods are 8.90% and 10.18%, respectively, demonstrating that the effectiveness of these two methods in reducing the error of the orientation results is limited. This is because both the OWT and OHHT methods treat the entire signal as the processing object, overlooking the differences in the degree of interference on the rotor signal at different sampling intervals, resulting in insufficient signal decomposition. Neither method could entirely eliminate the interference of abnormal jump data, making it difficult to restore the reverse torque information of the gyroscope accurately. In summary, while the OWT and OHHT methods have significant effects on improving signal smoothness, they still require further optimization in handling abnormal jumps and reducing the D value of orientation results.

After processing with the HSA-KS method, the average SD of the reconstructed signal is 16.14 × 10^−6^ A. The reason for the relatively large SD of the reconstructed signal is that this method does not process the data at the SSI, resulting in a significant amount of residual high-frequency white noise and some low-frequency noise interference in the reconstructed signal. Moreover, the D value gain rate of the HSA-KS method for the corresponding reconstructed signal is 26.76%. The HSA-KS method outperforms the OWT and OHHT methods regarding error gain because it directly eliminates the jump data, which are the most severely affected by external noise interference in the rotor signal. In summary, the HSA-KS method can reduce the D value of orientation results to a certain extent, but it still has insufficient performance in improving signal dispersion.

After processing with the MAF-ARIMA method, the average SD of the reconstructed signal is 5.44 × 10^−6^ A, significantly improving the smoothness of the reconstructed signal and the overall quality. Moreover, the D value gain rate of the reconstructed signal is 47.31%, indicating that this method can suppress abnormal jumps in data caused by instantaneous vibrations. The MAF-ARIMA method surpasses the HSA-KS approach by effectively eliminating abnormal jumps while comprehensively preserving critical reverse torque information, thereby enhancing the accuracy and reliability of the analysis. As shown in Table 1 and Table 2, the MAF-ARIMA method corresponds to the reconstructed signal with the minor average SD and D value. This experimental result fully validates the effectiveness of this method in noise reduction for rotor signals containing abnormal jumps.

It is worth noting that the capability of the MAF-ARIMA algorithm to effectively reduce noise largely depends on the presence of accurate and sufficient stable sampling data in the rotor signal. Through a comparative analysis of the original waveforms and their corresponding noise reduction results from multiple groups of abrupt rotor signals, we have observed that when the original rotor signal is subjected to severe instantaneous external disturbances, the overall data quality significantly deteriorates. In such cases, even with the application of the MAF-ARIMA method for noise reduction, the reconstruction error, although improved, still remains at a higher level compared to rotor signals with lesser degrees of disturbance, as evidenced by Groups 3, 5, and 6 in Table 2.

The main reasons for the larger errors in the noise-reduced reconstruction signals include the following:

(1) A high proportion of data from abnormal jump intervals. Severe external disturbances often result in the presence of three or more abnormal jump intervals in the rotor signal, leading to a higher proportion of abnormal jump data among all sampled data. This directly causes an insufficient amount of starting data for jump prediction, thereby limiting the effectiveness of the MAF-ARIMA algorithm.

(2) Low data quality in stable sampling intervals. For rotor signals severely affected by disturbances, the data in stable sampling intervals often exhibit higher standard deviations and mean instability. When these low-quality data are used as starting data, the inherent errors are propagated to the predicted data, thereby increasing the error in the reconstructed signal.

**Figure 14:**
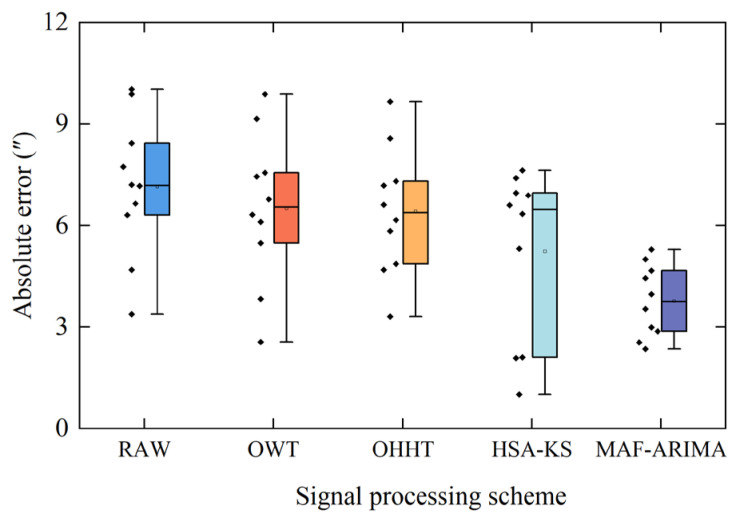
Box plot of D values corresponding to rotor signals reconstructed by different algorithms.

In addition, by comparing the box plots of the D values of the reconstructed signals processed by the four noise reduction algorithms (as shown in Figure 14), the D values corresponding to the MAF-ARIMA method are the most concentrated, further indicating the excellent stability of this method. Furthermore, based on the analysis of Table 2 and Figure 14, we can observe that when processing rotor signals containing jump data, the MAF-ARIMA algorithm has a significant advantage over the OWT and OHHT algorithms. This is because the OWT and OHHT algorithms fail to consider the time-varying characteristics exhibited by rotor signals under disturbance conditions and do not specifically treat jump data, making their overall noise reduction approach less effective for the noise reduction needs of jumping rotor signals. However, although the MAF-ARIMA method generally outperforms the HAS-KS algorithm in processing the results of most experimental groups of rotor signals, the processing results of the fourth, fifth, and ninth groups of experimental data reveal a trend in which the HAS-KS algorithm shows superior noise reduction effectiveness compared to the MAF-ARIMA method. This is primarily due to the HAS-KS algorithm’s more aggressive approach to detecting jump data, which involves removing as much abnormal data from the rotor signals as possible, thereby ensuring that the stable sampling data used for orientation measurement result calculations are of high quality. In contrast, the noise reduction effectiveness of the MAF-ARIMA method relies on accurate and sufficient stable sampling data, which has certain requirements for the quality and quantity of stable sampling data. Consequently, when processing rotor data containing a large number of scattered abnormal jumps, the noise reduction capability of the MAF-ARIMA method may be limited. Relatively speaking, its screening strategy is more conservative, which may result in it being less effective than the HAS-KS algorithm in certain situations.

## 5. Conclusions

This study proposes a MAF-ARIMA processing algorithm designed for rotor signals that contain abnormal jumps due to external instantaneous vibrations, thereby enhancing the accuracy of azimuth measurement results for magnetic suspension gyroscopes.

(1) To effectively reduce noise interference in rotor signals, we employ EMD adaptive decomposition to extract the effective components from the decomposition results and then apply MAF noise reduction to the SDC. Furthermore, by normalizing the maximum and minimum values and compensating for the mean, we correct the noise reduction results of the SDC_AJI_, ensuring consistency and continuity of data across different sampling intervals. This process enhances the stability and reliability of the denoised signal.

(2) To maintain the integrity of the rotor signal’s trend information, we utilize TDC_SSI_ as sample data for ARIMA prediction of missing TDC_AJI_ values. Additionally, we propose a bilateral linear weighting method to fuse multiple segments of prediction results, ensuring the accuracy and rationality of the predictions.

(3) To validate the effectiveness and stability of the proposed algorithm, we collected ten sets of rotor signals with abnormal jumps from the experimental area. The experimental results demonstrate that after processing with the MAF-ARIMA algorithm, the SD and D value of the reconstructed signals improved by 70.58% and 47.31%, respectively, significantly outperforming the other three noise reduction methods.

This study provides an efficient and practical method for noise reduction of gyroscope rotor signals, significantly reducing the interference of instantaneous vibrations on the orientation results of the torque feedback directional measurement system, and thereby promoting the development of tunnel breakthrough measurement technology. Additionally, during the comparison with other noise reduction algorithms, we have found that the noise reduction effectiveness of the MAF-ARIMA algorithm is significantly dependent on the quality of the initial data (i.e., stable sampling data). Due to the adoption of a relatively conservative strategy for identifying anomalous data, the algorithm’s capability to detect smaller magnitude or less frequent jump data in the rotor signal still requires further enhancement. Future research will focus on optimizing this aspect to improve the algorithm’s sensitivity and accuracy.

## Figures and Tables

**Table 1:** SD (×10^−6^ A) of reconstructed rotor signal corresponding to different methods.

Group	RAW	OWT [8]	OHHT [9]	HSA-KS [10]	MAF-ARIMA	Jumping Type
1	19.72	4.65	9.00	14.48	3.34	Endpoint
2	20.91	5.39	6.23	15.34	3.52	Middle
3	15.96	8.68	11.45	12.73	7.93	Middle
4	14.53	6.77	7.36	13.75	4.14	Middle
5	21.28	11.73	13.54	19.49	8.11	Endpoint
6	25.57	16.12	17.45	22.95	10.62	Endpoint
7	11.75	4.72	4.51	11.32	3.94	Middle
8	18.20	5.31	14.50	15.40	4.18	Endpoint
9	14.29	3.85	6.28	13.95	2.35	Endpoint
10	22.69	6.38	14.10	21.96	6.26	Middle
Mean	18.49	7.36	10.44	16.14	5.44	/
Improve	/	60.19%	43.53%	12.73%	70.58%	/

**Table 2:** D values (″) of reconstructed rotor signal corresponding to different methods.

Group	RAW	OWT [8]	OHHT [9]	HSA-KS [10]	MAF-ARIMA	Jumping Type
1	6.64	6.10	4.87	5.32	3.97	Endpoint
2	8.43	7.57	7.30	6.34	5.29	Middle
3	6.31	6.33	6.16	7.63	2.99	Middle
4	3.38	2.56	3.31	2.11	2.35	Middle
5	7.20	6.77	6.61	1.01	2.87	Endpoint
6	10.04	9.88	9.67	6.96	4.67	Endpoint
7	7.74	7.45	7.19	6.89	5.01	Middle
8	9.88	9.15	8.57	7.41	3.53	Endpoint
9	4.69	3.83	4.69	2.08	2.54	Endpoint
10	7.17	5.48	5.83	6.60	4.44	Middle
Mean	7.15	6.51	6.42	5.24	3.77	/
Improve	/	8.90%	10.18%	26.76%	47.31%	/

## Data Availability

The raw data supporting the conclusions of this article will be made available by the authors on request.
